# Notes and News

**Published:** 1889-10-12

**Authors:** 


### Notes and News.

Collected and Communicated.

The president, J. H. Horton, Esq., inspected the Horton Infirmary on Saturday, September 7th, accompanied by Mrs. Horton, Dr. Dundas Grant, and party. The Mayor and J. Harlock, Esq., J.P., attended for the Visiting Committee. The president expressed great gratification at the state of efficiency that he found prevailing in all the hospital arrangements.

The Open Scholarships in Arts at Guy's Hospital have this year been awarded to Mr. William S. Handley, Loughborough Grammar School and Mr. John Robert Steinhaeuser, Sutton Valence School. The Open Scholarships in Science have been awarded to Mr. John A. Howard, King's College and Guy's Hospital, and to Mr. Arthur H. Leete, University College, Aberystwith.

An amusing story comes from Paris of a poor boy brought to the hospital, wrapt in a blanket, and bound by chains. The director of the hospital learned from the people who brought the boy that he had about ten days ago been bitten by a dog which was supposed to have been mad. While playing with some other boys, he suddenly cried out, "Run away, or I shall bite you." Whether he did so for the sake of amusement and as a practical joke is not certain; but in any case the children took to their heels in terror, imagining that he was going mad. The parents then captured the lad and carried him off to the hospital as described. The boy was found to be perfectly sane and well.

It is greatly to be regretted that the Owens College authorities have not been able to avail themselves of the generous offer of the Whitworth Trustees. The authorities have dallied for a year, and are now surprised at receiving the following letter: "To Joseph Thompson, Esq., treasurer, Owens College.—My Dear Sir,—It is now rather more than a year since the Whitworth legatees made their offer to the Owens College in aid of the establishment and maintenance of a hospital in connection with the college. The legatees have been a little disappointed that means should not have been found for making use of that offer, and though they have been made aware of some difficulties in the way, have come to the conclusion that for present purposes it will be better for them to face the fact that there does not appear to be sufficient encouragement for them to proceed in this matter. We have decided, therefore, to withdraw our proposal of August, 1888, and I for my colleagues and myself do so now absolutely. I regret that I cannot at present qualify this retirement of ours by any other particular suggestions; and I only ask you to let me have a line of acknowledgment.—I am, my dear sir, yours very faithfully, (Signed) R. D. Darbishire."

The Hospitals Association enters upon its seventh session on the 16th October, when Dr. Bristowe, LL.D., F.R.S., will deliver the inaugural address on "The Work of the Association." The meeting will be held in the boardroom of Charing Cross Hospital at 8 p.m. All interested in the work of the hospitals are invited to attend. Tickets of admission may be obtained on application to the secretary, Norfolk House, Norfolk-street, Strand, W.C.

It is proposed to establish a Maternity Hospital at Eastbourne in connection with the Rescue Mission Home. Dr. Samuel Smith, in writing in favour of the project, says: "I have also learned some physiological facts I was not before acquainted with, namely, that in the cases of nearly all these poor girls rescued, sheltered, sympathised with and not coldly looked down on, there is an undoubted mental deficiency or peculiarity. They seem more gentle and simple than ordinary girls, and they also show distinct signs of nervous weakness."

We have sometimes been inclined to regard reports of institutions as rather dry reading; but one would wade through many wearisome pages to come across such a gem as the following, quoted from the last report of Torbay Hospital: "By the death of the late secretary, Mr. Pepprell, the hospital has lost an old and useful servant. It is satisfactory to report that his accounts were in order, and that, as far as is known, the hospital has sustained no loss through him." This ingenuous surprise at discovering an honest man in their midst scarcely speaks well for the general character of his contemporaries.

The ladies of Lancaster are to be congratulated on their energy and charity; they have collected £726 towards the new infirmary. It is proposed that the ladies bear the expense of the children's wing. At a recent meeting Mr. Satterthwaite, as the treasurer of the building fund, said he felt very much indebted to the ladies who had come forward and raised such an amount as they had done. The building fund receipts amounted to £11,869, to which must be added the handsome contributions raised by the ladies, amounting to £725, making a total of £12,594; £726 had also been raised for the endowment fund. They reckoned that the premises they now occupied were worth £2,000, so that they had now standing to the credit of the building fund £14,594. On behalf of the committee, he wished to thank the ladies for the result of their efforts.

We commend to all over-sensitive persons, and especially to women, Dr. W. Collier's article on sensation in animals, in the *Nineteenth Century*. The awful treatment of animals witnessed on the continent, and the more awful tales told by anti-vivisectors, are simply torture to many people cursed with too finely strung nerves. By the bye, we are inclined to believe that anti-vivisectionists cause more pain in the animal world by circulating their ghastly pamphlets among hysterical women, than do medical men who are obliged to perform experiments occasionally. Dr. W. Collier distinctly proves in a popular manner the comparative insensibility of animals. With regard to fish Dr. Collier says: "Every fisherman has his story to tell either of himself or of a friend having hooked a fish with another hook recently embedded in its flesh. The great difficulty of killing some fish would prove at any rate that their nervous system was not over-sensitive to shock, while the extreme smallness of their brains would strengthen our belief in their want of sensitiveness, for whereas the proportion of the brain of man to the rest of his body is about as one to sixty, the proportion in fishes is about one to three thousand. In man and animals the skin is certainly the most sensitive tissue of the body; we can hardly imagine that the scales which cover the bodies of fishes are equally sensitive."

The following vacancies are announced this week, the amount of the salary in each case being given after the name of the institution:

**Resident Medical Officers.**

Liverpool Infirmary for Children. House Surgeon.—£85, board, &c.
Leeds General Infirmary. Obstetrical Officer, Two House Surgeons, and One House Physician.
Clayton Hospital. House Surgeon.—£120, rooms, &c.
Dorset County Asylum. Assistant Medical Officer.—£120, board, &c.
Lancashire County Asylum. Assistant Medical Officer.—£105, board, &c.
Hants County Asylum. Junior Assistant Medical Officer.—£100, board, &c.
Sheffield Infirmary. House Surgeon.—£120. Assistant House Surgeon.—£80, board, &c.
Paddington Green Children's Hospital. House Surgeon.—£50, board, &c.
Jersey Infirmary. Medical Officer.—£120, rooms, &c.
Hospital for Sick Children, Great Ormond-street. Medical Officer.—£50, board, &c.

**Non-Resident Medical Officers.**

Stokesley Union. Medical Officer.—£54.
Dundee University College. Professor of Physiology.—£350.
London Lock Hospital. Physician and Surgeon to Out-Patients.
Manchester Owens College. Junior Demonstrator in Physiology.—£100.

The annual report of Providence Hospital, St. Helens, states that during the past year there has been an increase of nearly one hundred patients, and the managers and committee see with joy the continued usefulness of the hospital. Kind friends and supporters have increased in number. Many of these friends have employed themselves in making small collections from those unable to contribute largely, and thus the considerable sum of £158 0s. 8½d. has been added to the funds. Had not the number of patients increased, the deficit would have been paid off. Even as it is this has diminished. The hospital is nursed and managed by the sisters of Brentford Convent.

The governors of Lowestoft Hospital have held their annual meeting, and their report is one long cry for funds. The present income from funded capital, annual subscriptions, and church collections, which constitute the entire reliable receipts of the hospital, amount together to about £1,000 a year, and the expenditure to about £1,250. If the present state of things should continue, and there is certainly no hope that the expenses will be diminished, only two courses will be left to the committee—either to draw upon the capital or limit the number of in-patients by partially closing the wards. The committee feel they have only to put this matter clearly before the public to ensure their sympathy and help towards maintaining the institution. During last year there have been 16 patients, on an average, each day in the wards of the hospital.

It is interesting to note that, some years ago, when a "pioneer" case of removal of tumour from the brain was performed at the Hospital for Epilepsy and Paralysis, the wrath of the anti-vivisectionists was greatly stirred. One letter received by the house surgeon of the hospital began thus: "I have seen several accounts in local papers of your last wonderful cure on brain; will you produce the patient cured, not till then will you vivisectors be believed. Gamgee, Ringer, and others of your blood-stained fraternity have opened the eyes of the public as to the horrible doings in your dens of torture." There were pages and pages of this vulgar abuse, and it is merely quoted now that time has made common the operation complained of, an operation which is constantly successful and has saved many lives. The letter proves how distorted men's minds can become when narrowed to hold one idea only.

Newcastle College of Medicine has been formally opened by the Mayor. The foundation-stone of the present buildings was laid by the Duke of Newcastle in 1887. These buildings have cost about £30,000, and a debt remains of £10,000. The college has no endowment, its funds being derived exclusively from students' fees. Dr. Heath, president of the college, occupied the chair at the opening proceedings, and handed a cheque to the treasurer for 1,000 guineas.

Scarlet fever is steadily increasing, and the Asylums Board has had to open more hospitals. The returns presented last Saturday showed that there was an increase of 136 in the total number of patients remaining under treatment as compared with the previous fortnight. There had been 361 direct admissions in the two weeks, 47 deaths, and 178 discharges, and there were on Friday night 1,456 cases under treatment, of which 1,250 were scarlet-fever cases, 114 diphtheria cases, 88 enteric cases, one case of typhus, and three cases of other diseases.

The October magazines contain much of medical interest. In *Harper's*, Dr. Keen discourses on "Recent Progress in Surgery." Several amazing triumphs of abdominal surgery are recorded, and these are supplemented by some extraordinary instances of operations connected with the brain. Reference is made to a paper recently published by Mr. Horsley, which includes an account of ten operations on the brain, "in which, without anything on the exterior to indicate its situation, the site of the disease was correctly located in all, nine of the patients recovering after operation."—In *Temple Bar* is an interesting story, called "Dr. Gabriel's Experiment." Dr. Gabriel has suddenly lost a young and deeply-loved wife. Though he becomes at times aware of her spiritual presence he can never succeed in recalling her face. That the presence is real he is convinced, from the agitation shown by some goldfish. This gives him the suggestion for his experiment. He considers that as there are rays beyond the red and violet ends of the prism invisible to our eyes, some of which have yet been proved to have an effect upon ants, the eyes of the goldfish may be constituted so as to perceive the spiritual presence which was hidden from him. How his experiment answers we must not state here.

The suicide of Sir Wm. Tindal Robertson will be a great shock to many of his personal friends in the medical profession. He was a man who had shown conspicuous bravery under affliction, notably the loss of his eyesight, and that he should finally have given way before the force of circumstances is inexpressibly sad. The eldest son of the late Frederick Fowler Robertson, of Bath, Sir Tindal was educated at King Edward the Sixth's Grammar School, and entered the medical profession as house pupil to Mr. H. P. Robarts, of Great Coram-street, London. He studied at University College Hospital, matriculated at London University, and successively became a Licentiate of the Apothecaries' Company, a Member of the Royal College of Surgeons, Junior Resident Medical Officer at Middlesex Hospital, Doctor of Medicine at the University of Edinburgh, and a Fellow of the College of Physicians. He commenced practice as a physician in Nottingham in 1854, where he resided till the loss of his sight. In 1857, when the British Medical Association held its congress at Nottingham, he was elected to deliver the address on medicine, taking as his subject "The Rise and Progress of Sanitary Science," whilst at the meeting of the same association in 1865 he introduced the question, "Why are sanitary measures not always followed by a reduction of mortality?" Among other literary works he furnished to Mr. Churchill's "Photographs of Eminent Medical Men" the analytical notices of their works.

